# Paramagnetic NMR Investigation of Dendrimer-Based Host-Guest Interactions

**DOI:** 10.1371/journal.pone.0064722

**Published:** 2013-06-10

**Authors:** Fei Wang, Naimin Shao, Yiyun Cheng

**Affiliations:** 1 Shanghai Key Laboratory of Regulatory Biology, School of Life Sciences, East China Normal University, Shanghai, People's Republic of China; 2 Shanghai Key Laboratory of Magnetic Resonance, Department of Physics, East China Normal University, Shanghai, People's Republic of China; Brandeis University, United States of America

## Abstract

In this study, the host-guest behavior of poly(amidoamine) (PAMAM) dendrimers bearing amine, hydroxyl, or carboxylate surface functionalities were investigated by paramagnetic NMR studies. 2,2,6,6-Tetramethylpiperidinyloxy (TEMPO) derivatives were used as paramagnetic guest molecules. The results showed that TEMPO-COOH significantly broaden the ^1^H NMR peaks of amine- and hydroxyl-terminated PAMAM dendrimers. In comparison, no paramagnetic relaxation enhancement (PRE) was observed between TEMPO-NH_2_, TEMPO-OH and the three types of PAMAM dendrimers. The PRE phenomenon observed is correlated with the encapsulation of TEMPO-COOH within dendrimer pockets. Protonation of the tertiary amine groups within PAMAM dendrimers plays an important role during this process. Interestingly, the absence of TEMPO-COOH encapsulation within carboxylate-terminated PAMAM dendrimer is observed due to the repulsion of TEMPO-COO- anion and anionic dendrimer surface. The combination of paramagnetic probes and ^1^H NMR linewidth analysis can be used as a powerful tool in the analysis of dendrimer-based host-guest systems.

## Introduction

Dendrimers are a new class of nanoscopic containers and delivery devices [1], [2], [3], [4]. They have unique molecular architectures, spherical shapes, interior cavities, excellent monodispersity, and high density of surface functionalities [5], [6], [7], [8], [9], [10], [11]. Dendrimers have high solubility in various organic solvents and water, allowing miscellaneous modifications on dendrimer surface and in-depth characterization by different techniques [4]. These physicochemical properties render dendrimers the applications in host-guest systems [12], [13]. Tomalia *et al*. first reported the diffusion of aspirin molecules into the interior cavities of poly(amidoamine) dendrimers by NMR relaxation measurements [14]. Meijer *et al.* synthesized a dendritic box by modification of poly(propylene imine) (PPI) dendrimer surface with 9-fluorenylmethyloxycarbonyl (FMOC)-protected amino acids and the dendritc box was able to encapsulate guest molecules such as Rose Bengal and *p*-nitrobenzoic acid [15], [16], [17]. The release of *p*-nitrobenzoic acid from the dendritic box can be triggered by removing FMOC protection, while Rose Bengal cannot diffuse out of the dense shell due to its larger size than *p*-nitrobenzoic acid [17]. In a separate study by the Meijer group, palmitic acid modified PPI dendrimers were successfully used for the extraction of anionic xanthene dyes from aqueous solution [18]. These pioneer studies significantly promote the interests of scientific community in dendrimer-based host-guest systems [12], [19], [20], [21].

Our group systematically investigated PAMAM and PPI dendrimer-based host-guest systems using several NMR techniques in the past five years [21], [22], [23], [24], [25], [26]. NMR techniques have proved to be powerful and informative tools in the characterization of dendrimers and dendrimer/guest complexes [12]. Dendrimers have successive repeat units, well-defined branched structures, highly symmetric frameworks, and spherical shapes like proteins [27]. Repeat unit means rapid chemical shift assignment of the dendrimer protons and much simplified NMR spectrum [12]. Highly symmetric framework and well-defined branched structures allow us to predict the location and orientation of guest molecules within the interior pockets of dendrimers by nuclear Overhauser effect spectroscopy (NOESY) [22], [25]. Spherical shape encourages us to characterize the size and morphology of dendrimers and dendrimer/guest complexes or aggregates using diffusion NMR techniques such as pulsed gradient spin echo (PGSE) and diffusion ordered spectroscopy (DOSY) [26], [28]. Besides, the rapid technological development in high-field NMR spectroscopy and use of cryogenic probe system significantly increase the sensitivity in NMR analysis, and reduce the time and sample concentration needed in an NMR experiment.

When a paramagnetic probe such as nitroxide spin label is in spatial proximity with a host molecule, magnetic dipolar interactions between the unpaired electrons of the paramagnetic probe and the nucleus of interest will result in an increase in nuclear relaxation rates, which is defined as paramagnetic relaxation enhancement (PRE) effect [29]. In comparison with NOE, where the effect is limited to a short range within 5–6 Å, the PRE method can be used to get distance information in the range of 15–24 Å for nitroxide spin label, owing to the large magnetic moment of the unpaired electron [30]. Distance between the spin label and the nucleus of interest can be determined from the increased *R*_2_ relaxation rates, allowing quantitatively interpretation of the PRE effect [31]. As a result, PRE NMR is becoming a sensitive and powerful tool to produce long-range site-specific constraints that can complement NOE constrains [29], [32].

**Figure 1:**
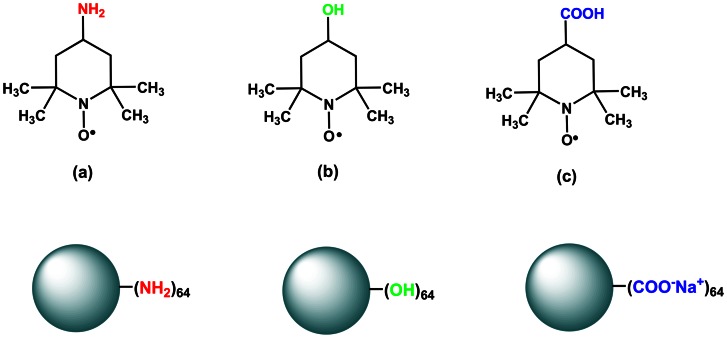
Molecular structures of the three nitroxide spin probes (a) TEMPO-NH_2_ (b) TEMPO-OH (c) TEMPO-COOH and structure chart of PAMAM dendrimers with three types of surface groups.

**Figure 2:**
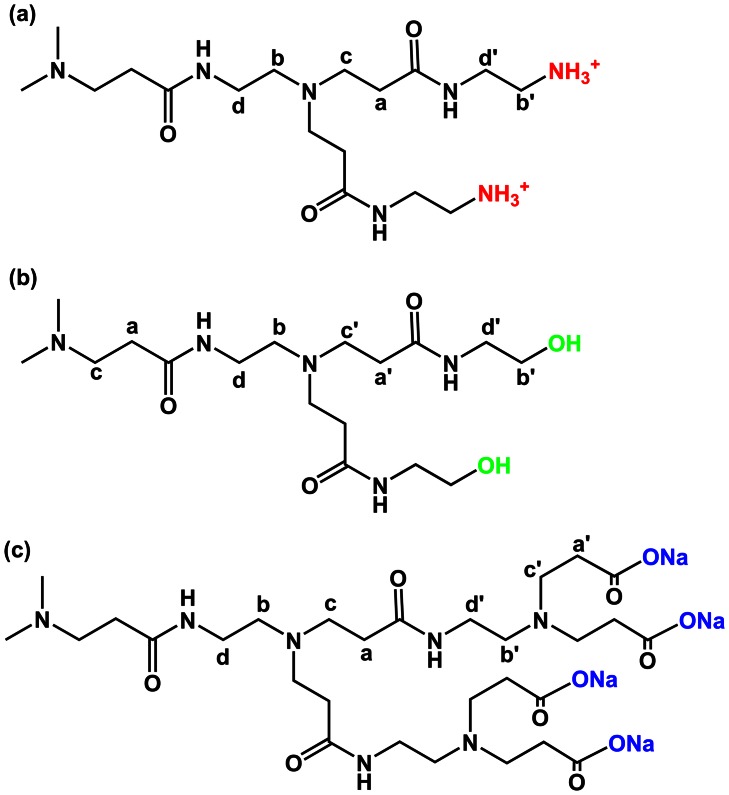
Molecular structures and proton labeling of PAMAM dendrimers. Amine-ternimated (a), hydroxyl-terminated (b) and carboxylate-terminated (c) PAMAM dendrimers.

In dendrimer-based host-guest systems, if a paramagnetic probe is encapsulated within the dendrimer interior pockets, the PRE effect will lead to decreased NMR signal intensities and increased peak linewidths of dendrimer protons close to the paramagnetic probe in a ^1^H NMR spectrum, which is an important supplement to the NOESY techniques in the investigation of dendrimer-based host-guest systems [29]. Previously, Newkome *et al*. investigated dendrimer structure by different NMR techniques using paramagnetic cobalt (II) as a probe [33], [34]. In this study, 2,2,6,6-tetramethylpiperidinyloxy (TEMPO) derivatives were used as paramagnetic guests (**Fig. 1**). PAMAM dendrimers bearing hydroxyl, primary amine, and carboxylate groups were used as model hosts (**Fig. 2**). Though the interactions of TEMPO derivatives and PAMAM dendrimers were previously investigated by Turro and Tomalia *et al*, they use an electron spin resonance (ESR) technique to analyze the dendrimer/TEMPO complexes [35], [36]. The major goal of this study is to reveal the host behaviors of PAMAM dendrimers with different surface functionalities by PRE NMR.

## Experimental

### Materials

Generation 4 (G4) ethylenediamine (EDA)-cored and primary amine-terminated PAMAM dendrimer (G4-NH_2_), G4 EDA-cored and hydroxyl-terminated PAMAM dendrimer (G4-OH), and G3.5 EDA-cored and carboxylate-terminated PAMAM dendrimer (G3.5-COONa) were purchased from Dendritech (Midland, MI). 4-amino-TEMPO (TEMPO-NH_2_) and 4-carboxy-TEMPO (TEMPO-COOH) were purchased from Sigma-Aldrich (St. Louis, MO). 4-hydroxy-TEMPO (TEMPO-OH) was obtained from Aladdin Chemistry Co. Ltd (Shanghai, China). Deuterium oxide (D_2_O) was purchased from Beijing Chongxi High-Tech Incubator Co. Ltd. (Beijing, China). Dioxane and deuterated dimethyl sulfoxide (d6-DMSO) were purchased from Aladdin Chemistry Co. Ltd (Shanghai, China). Dendrimers were received in methanol and the solvents were distilled to obtain the products as white gels. All other chemicals were used as received without further purification.

### Sample preparation

G4-NH_2_, G4-OH, and G3.5-COONa were dissolved in D_2_O at a concentration of 20 mg/mL. TEMPO-NH_2_, TEMPO-COOH, and TEMPO-OH were dissolved in d6-DMSO at a concentration of 5 mg/mL. The stock solutions were stored at 4°C before NMR studies. The dendrimer/TEMPO complex solutions were prepared at different TEMPO/dendrimer molar ratios in 500 µL D_2_O and d6-DMSO mixtures (V/V, 80/20). Dendrimer concentrations in the complex solutions are fixed at 1 mg/mL and the molar ratios of TEMPO/dendrimer are 0, 1, 2, 4, 8, 12, 16, 24, 32, 40, 48, and 64, respectively. Each sample contains a certain amount of dioxane which was used as internal standard. The samples were sonicated for 2 h before NMR analysis and the pH condition of each sample was measured immediately after the NMR experiments.

### NMR analysis

^1^H NMR spectra of the PAMAM dendrimer and TEMPO complex solutions were obtained on a Varian 699.804 MHz NMR spectrometer at 298.2±0.1 K. The peak linewidths in ^1^H NMR spectrum were measured using the VNMRJ software.

## Results and Discussion

### Interactions of TEMPO-NH_2_ and TEMPO-OH with three types of PAMAM dendrimers

**Figure 3:**
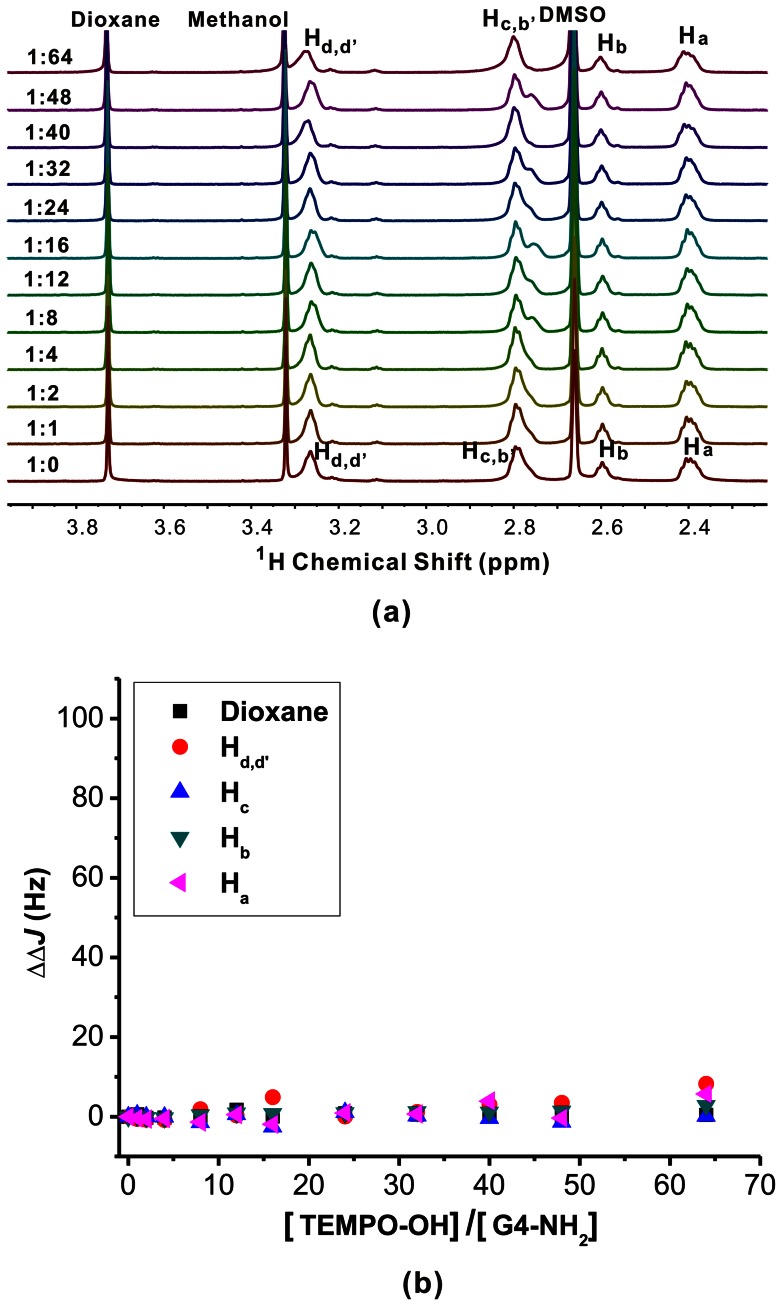
The effect of TEMPO-OH on the signal intensity of G4-NH_2_. (a) ^1^H NMR spectra of the G4-NH_2_/TEMPO-OH complexes in D_2_O/d6-DMSO solutions (80/20, V/V), the molar ratio of TEMPO-OH and G4-NH_2_ ranges from 0 to 64. (b) The linewidth variations of G4-NH_2_ peaks in ^1^H NMR spectra during the titration experiment.

The host-guest chemistry of TEMPO-NH_2_ and TEMPO-OH with PAMAM dendrimers bearing amine, hydroxyl, and carboxylate groups were investigated by ^1^H NMR. G4-NH_2_, G4-OH, and G3.5-COONa PAMAM dendrimers were used because these dendrimers have the same numbers of surface functionalities (64) and similar molecular size. Besides, these PAMAM dendrimers were reported to have interior hydrophobic pockets and open surface structures, which are excellent candidates in host-guest systems [3], [5]. As shown in Fig. 3, G4-NH_2_ has four ^1^H NMR peaks: H_d, d'_ at 3.26 ppm, H_c, b'_ at 2.79 ppm, H_b_ at 2.60 ppm, and H_a_ at 2.40 ppm, respectively. The peak at 3.72 ppm corresponds to the internal standard dioxane, while peaks at 3.32 ppm and 2.66 ppm arise from the residual methanol and DMSO, respectively. Both the chemical shift and linewidth of the G4-NH_2_ peak were scarcely affected by the addition of TEMPO-OH. Previous studies have demonstrated that ionic binding of anionic guests on the surface of amine-terminated PAMAM dendrimer shifts the resonance signals of dendrimer surface (H_b'_ and H_d'_) to higher frequencies, and hydrophobic encapsulation of guests within dendrimer interior pockets shifts the signals of dendrimer interior (H_a∼d_) to lower frequencies. [12] The absence of H_a∼d_ signal shifts in Fig. 3 indicates the absence of ionic binding and hydrophobic encapsulation. In addition, TEMPO-OH is a paramagnetic guest molecule. If TEMPO-OH molecules are encapsulated within the G4-NH_2_ interior cavities via hydrophobic or ionic interactions, the PRE effect will lead to significant enhancement of linewidths of G4-NH_2_ resonance signals. However, no significant variation in linewidth of G4-NH_2_ signals during the addition of TEMPO-OH in Fig. 3b suggests the absence of TEMPO-OH encapsulation within G4-NH_2_. Similarly, when TEMPO-NH_2_ was added into G4-NH_2_, no significant change in the ^1^H NMR spectrum of G4-NH_2_ was observed ( Fig. S1). The only difference is the slight shifts of H_b'_ and H_d'_ to lower frequency when increasing TEMPO-NH_2_/G4-NH_2_ molar ratio. During the addition of TEMPO-NH_2_ into G4-NH_2_, the pH value of the dendrimer solution increases from 8.72 to 9.59 when the molar ratio of TEMPO-NH_2_/G4-NH_2_ reaches 64. Deprotonation of the NH_3_^+^ groups (pKa∼10.0) on G4-NH_2_ surface well explains the slight shifts of H_b'_ and H_d'_ signals in Fig. S1. The absence of G4-NH_2_ linewidth variation during TEMPO-NH_2_ titration also confirms the absence of TEMPO-NH_2_ encapsulation within G4-NH_2_.

**Figure 4:**
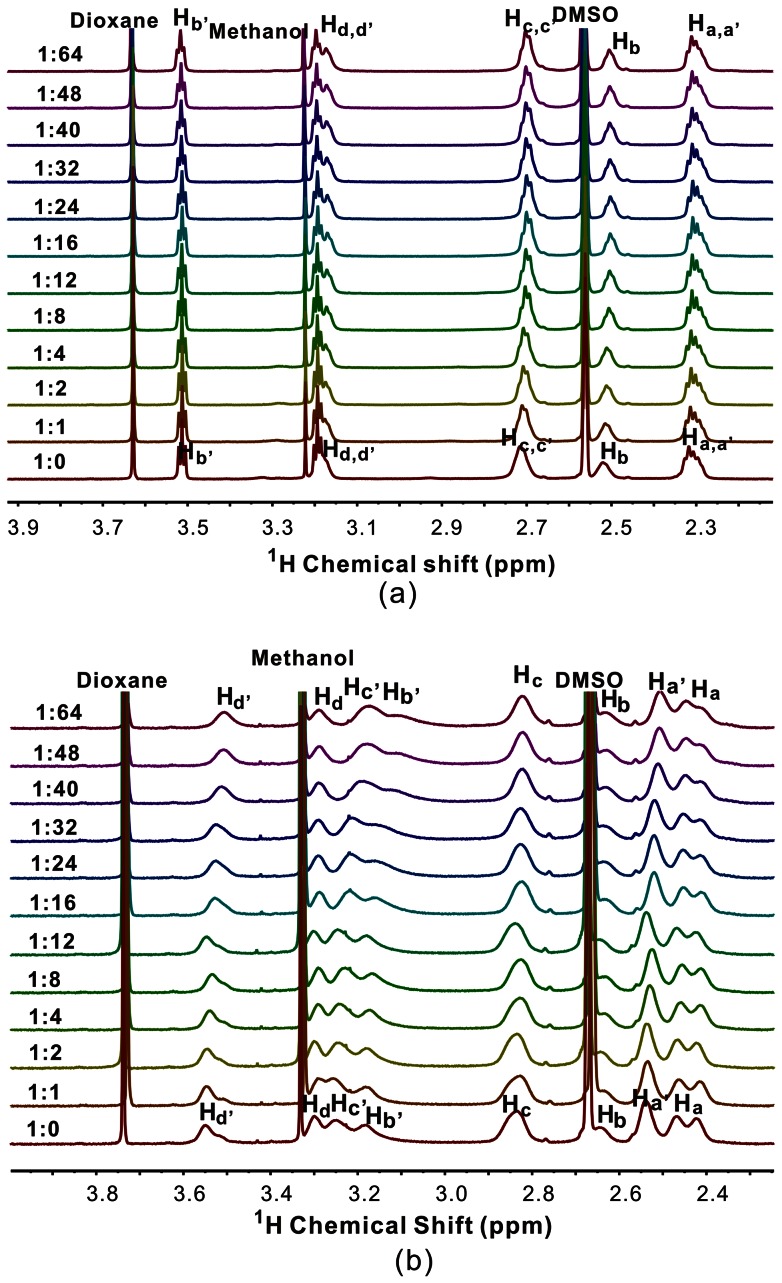
The effect of TEMPO-NH_2_ on the signal intensity of G4-OH and G3.5-COONa. ^1^H NMR spectra of the G4-OH/TEMPO-NH_2_ (a) and the G3.5-COONa/TEMPO-NH_2_ (b) complexes in D_2_O/d6-DMSO solutions (80/20, V/V), the molar ratio of TEMPO-NH_2_ and dendrimer ranges from 0 to 64.

Similar phenomenons are observed between TEMPO-NH_2_ (TEMPO-OH) and G4-OH (G3.5-COONa) (Fig. 4 and Fig. S2). These results suggest that no host-guest interaction occurs between TEMPO-NH_2_ (TEMPO-OH) and the three types of PAMAM dendrimers. TEMPO-NH_2_ (171 Da) and TEMPO-OH (172 Da) have dendrimer pocket-matched sizes. Our previous studies have reported successful encapsulations of much larger guests such as sodium dodecyl sulfate (288 Da), dexamethasone 21-phosphate (516 Da), and Congo red (696 Da) within G4-NH_2_ PAMAM dendrimer [21], [25], [37]. Therefore, the failure of TEMPO-NH_2_ and TEMPO-OH encapsulation within PAMAM dendrimers should not be attributed to size limitations of the guests, but probably to the reason that PAMAM dendrimer pockets are not hydrophobic enough to encapsulate TEMPO-NH_2_ or TEMPO-OH in d6-DMSO/D_2_O (20%/80%, V/V) via hydrophobic interactions.

### Interactions of TEMPO-COOH with G4-NH_2_ and G4-OH PAMAM dendrimers

**Figure 5:**
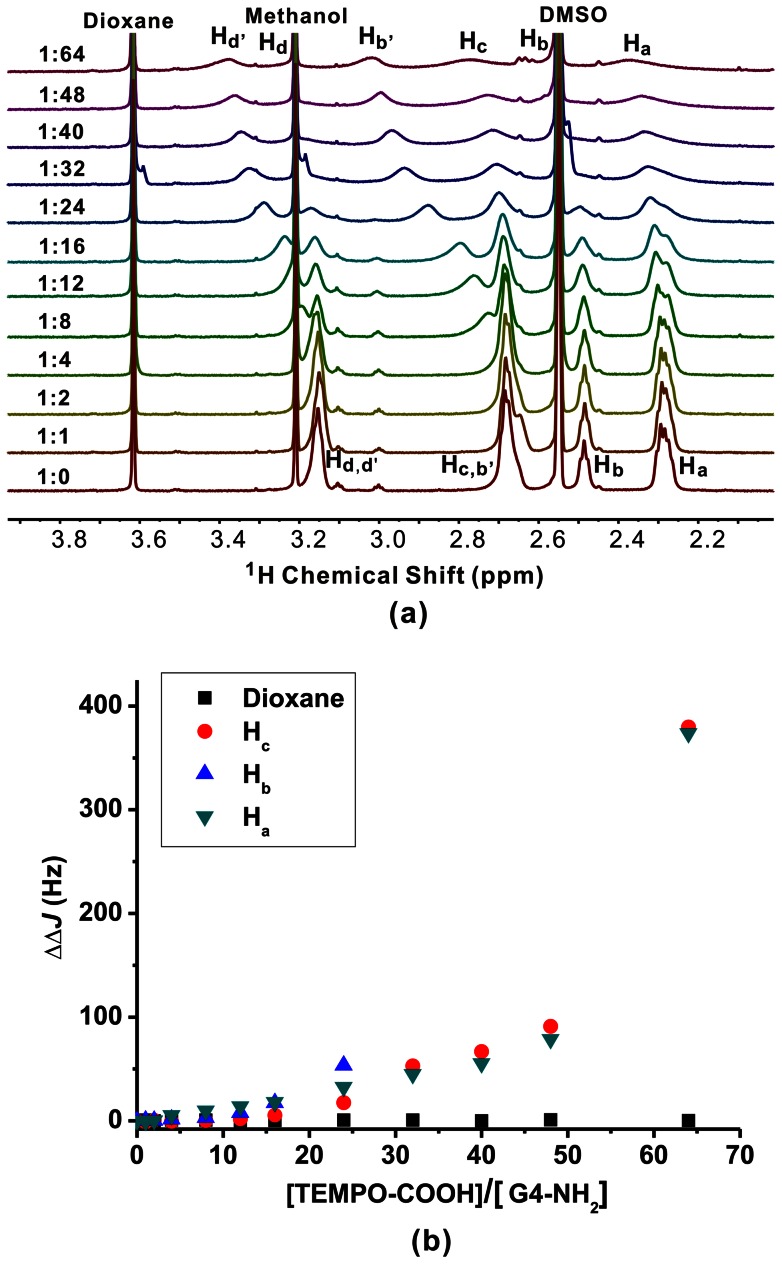
Increased concentration of TEMPO-COOH decreases the signal intensity of G4-NH_2_. (a) ^1^H NMR spectra of the G4-NH_2_/TEMPO-COOH complexes in D_2_O/d6-DMSO solutions (80/20, V/V), the molar ratio of TEMPO-COOH and G4-NH_2_ ranges from 0 to 64. (b) The linewidth variations of G4-NH_2_ peaks in ^1^H NMR spectra during the titration experiment.

As shown in Fig. 5, signal intensities of all the G4-NH_2_ resonance signals (H_a∼d_, H_b'_ and H_d'_) are gradually decreased with increasing TEMPO-COOH/G4-NH_2_ molar ratios. Also, the linewidths of the signals are much broader after the addition of TEMPO-COOH molecules. This phenomenon can be recognized as an evidence of TEMPO-COOH encapsulation within G4-NH_2_. One may argue that decreased molecular mobility or increased molecular size also contribute to increased peak linewidth. In the system of G4-NH_2_ and TEMPO-COOH, this effect is not significant as compared to the PRE effect. Take G4-NH_2_ for an example, its diffusion coefficient is slightly changed after the addition of 8 or 16 molar ratios of TEMPO-COOH (Fig. S3). When more TEMPO-COOH were added (molar ratio of 32 or 64), the peak intensities of G4-NH_2_ are extremely low due to the PRE effect and the diffusion coefficients of G4-NH_2_ are not available using pulsed gradient spin-echo (PGSE) NMR. In this case, 32 molar ratios of deoxycholate which was proved to interact with G4-NH_2_ were used instead of TEMPO-COOH. [24] Again, only a slight change (∼3%) on the diffusion coefficient of G4-NH_2_ was observed before and after the addition of deoxycholate (Fig. S3). These results suggest that the increased peak linewidths in Fig. 5 are mainly caused by the PRE effect of TEMPO-COOH encapsulated within G4-NH_2_ pockets rather than the increased dendrimer size after TEMPO-COOH addition.

Besides variations in signal intensity and linewidth, shifts of H_b'_ and H_d'_ resonance signals to higher frequencies in Fig. 5 were observed. This is due to ionic interactions between deprotonated TEMPO-COOH and cationic charged G4-NH_2_ surface [38], [39]. The pH value of TEMPO-COOH/G4-NH_2_ solution decreases to 5.61 when the molar ratio of TEMPO-COOH and G4-NH_2_ reaches 64. As the pKa values of surface amine and interior tertiary amine groups of PAMAM dendrimers are around 10.0 and 6.5 respectively [40]. All the surface amine groups and partial interior tertiary amine groups of G4-NH_2_ should be positively charged when the molar ratio of TEMPO-COOH and G4-NH_2_ is above 40 (pH 6.81). In such a complex solution, TEMPO-COOH is negatively charged and can be either bound on the surface or encapsulated within the interior of G4-NH_2_ via ionic interactions. To exclude the possibility that the decreased signal intensity and the broadened peak linewidth are caused by amine protonation rather than the PRE effect, we titrated G4-NH_2_ with acetic acid. As shown in Fig. S4, almost no change in signal intensity and peak linewidth of H_a–d_ are observed. The changes in linewidths of H_b'_ and H_d'_ in Fig. S4 can be explained by exchanges of NH_3_^+^ and NH_2_ on G4-NH_2_ surface during the addition of acetic acid. We also used NOESY to prove the encapsulation of TEMPO-COOH within G4-NH_2_. As TEMPO-COOH is a paramagnetic molecule, the PRE effect broadens both the G4-NH2 and the TEMPO-COOH peaks as shown in Fig. 5 which makes it difficult to detect the cross-peaks of between G4-NH_2_ and TEMP-COOH using a NOESY method. In this case, we treated the TEMPO-COOH with 12 N HCl to scavenge the TEMPO radical. The resulting guest was complexed with G4-NH_2_ at a molar ratio of 32∶1 and the complex was analyzed by ^1^H-^1^H NOESY at a mixing time of 300 ms. As shown in Fig. S5, weak NOE cross-peaks between the methyl groups of the guest and the methylene protons (H_a, c_) of G4-NH_2_ were observed, indicating the encapsulation of the TEMPO-COOH derivative within G4-NH_2_. This result indicates that the PRE NMR method can be used as an alternative method to NOESY in the investigation of inclusion structures. The PRE method using ^1^H NMR to probe host-guest interaction is more facile and sensitive than the NOESY method.

**Figure 6:**
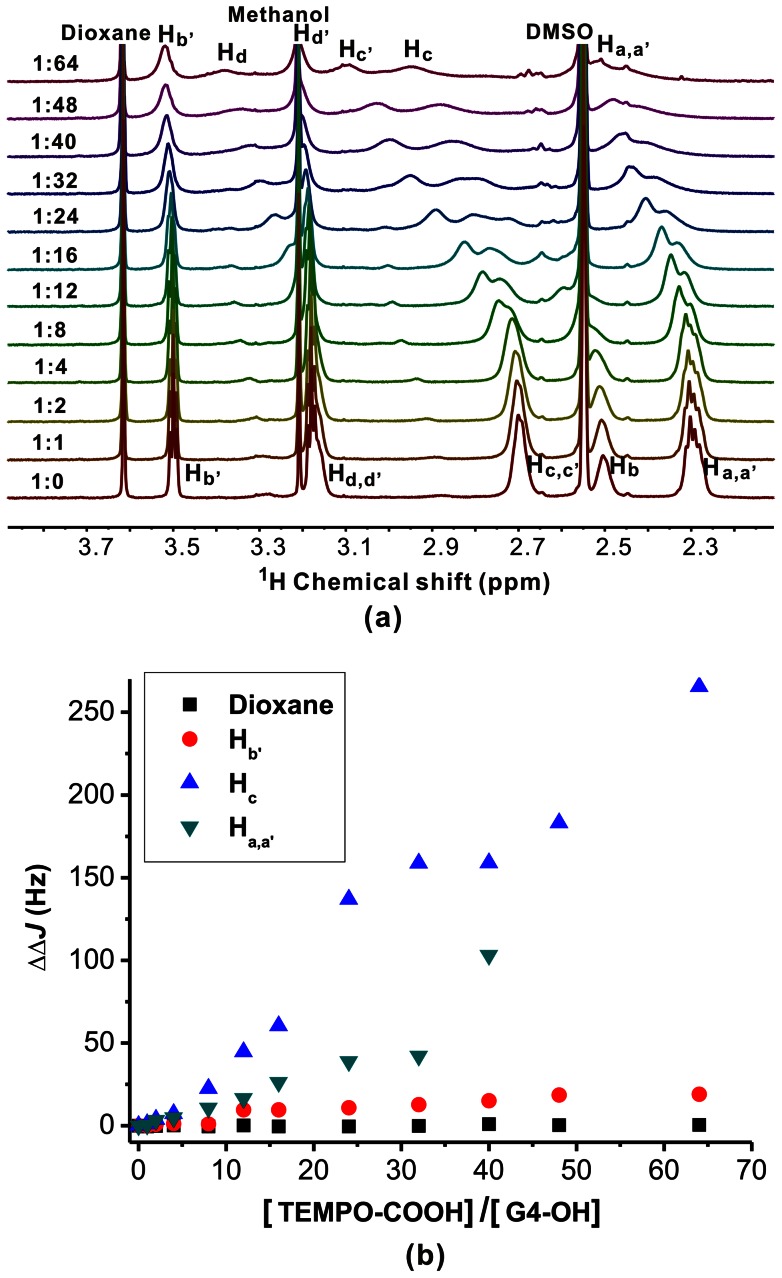
The effect of TEMPO-COOH on proton signal intensity of G4-OH. (a) ^1^H NMR spectra of the G4-OH/TEMPO-COOH complexes in D_2_O/d6-DMSO solutions (80/20, V/V), the molar ratio of TEMPO-COOH and G4-OH ranges from 0 to 64. (b) The linewidth variations of G4-OH peaks in ^1^H NMR spectra during the titration experiment.

When G4-OH was titrated with TEMPO-COOH or acetic acid, the protonation of the interior tertiary amine groups of G4-OH also caused the broadening of resonance signals for adjacent methylene protons such as H_a–d_ (Fig. 6 and Fig. S6). This makes it difficult to analyze the PRE effect in the system. However, the variations of linewidth for H_b'_ in the samples titrated with TEMPO-COOH are much higher than that in the samples titrated with acetic acid (Fig. S6), indicating the presence of PRE effect in TEMPO-COOH/G4-OH. Since the surface of G4-OH is non-charged, no ionic interaction occurs between TEMPO-COOH and G4-OH surface. TEMPO-COOH molecules should be at least encapsulated within the outermost layer of G4-OH dendrimer. Interior methylene protons (H_a–d_) of G4-OH dendrimer exhibit significant higher frequency shift, while surface methylene protons of G4-OH such as H_b'_ and H_d'_ are slightly shifted, suggesting quaternization of the interior tertiary amine groups of G4-OH (pH ranges from 8.11 to 5.10). The formation of inclusion complexes of TEMPO-COOH with G4-NH_2_ or G4-OH should be driven by ionic interactions rather than hydrophobic interactions. This property is distinct from previous dendrimer/guest inclusion complexes such as PAMAM dendrimers with mycophenolic acid, [12] sodium deoxycholate and sodium dodecyl sulfate, [24] and phenylbutazone [22], in which the encapsulations are driven by hydrophobic interactions.

### Interaction of TEMPO-COOH with G3.5-COONa

**Figure 7:**
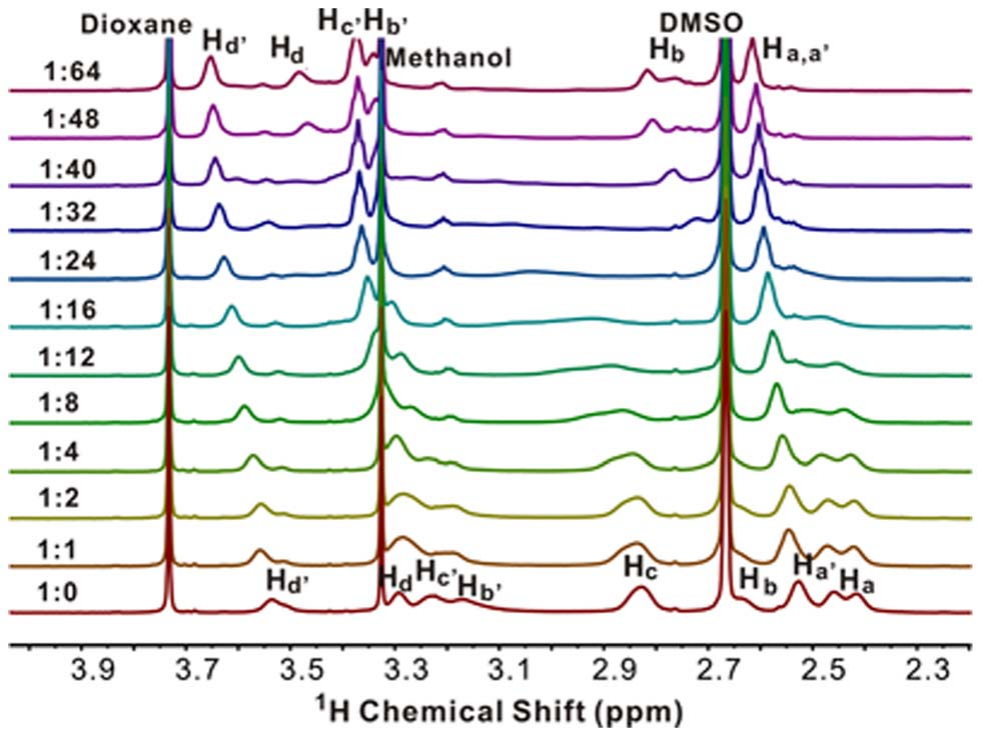
^1^H NMR spectra of G3.5-COONa/TEMPO-COOH complexes. The molar ratio of TEMPO-COOH and G3.5-COONa ranges from 0 to 64. The solvent is D_2_O/d6-DMSO (80/20, V/V).

**Figure 8:**
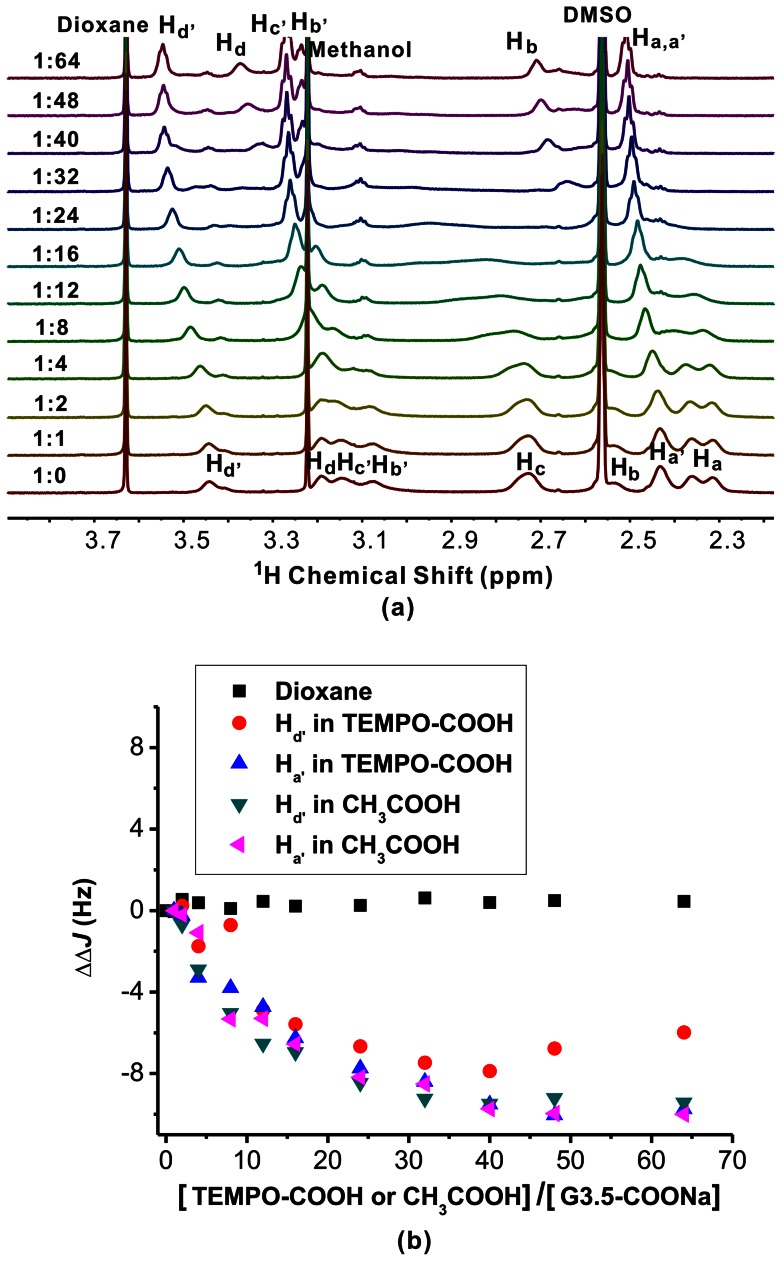
Comparison of TEMPO-COOH and acetic acid on the signal intensity of G3.5-COONa. (a) ^1^H NMR spectra of the G3.5-COONa/acetic acid complexes in D_2_O/d6-DMSO solutions (80/20, V/V), the molar ratio of acetic acid and G3.5-COONa from 0 to 64. (b) The linewidth variations of G3.5-COONa peaks in ^1^H NMR spectra during the titration of G3.5-COONa with TEMPO-COOH and acetic acid.

As shown in Fig. 7, titration of G3.5-COONa with TEMPO-COOH leads to higher frequency shifts of all the dendrimer protons. During this period, the pH value of the complex solution gradually decreases from 9.04 to 5.45, which means partial protonation of interior tertiary amine groups within G3.5-COONa. Interestingly, the signal intensities of peaks H_a', b', c', d'_ increase during the addition of TEMPO-COOH. Meanwhile, the linewidths of these peaks become lower. This result is not in accordance with those observed in TEMPO-COOH/G4-NH_2_ and TEMPO-COOH/G4-OH. This phenomenon can be explained by the equilibrium equation in Fig. S7. Due to the presence of carboxylate groups on G3.5-COONa surface, the tertiary amine group located on the outermost layer has a much higher pKa value (8.0∼9.0) than the interior teriary amine groups (pKa∼6.5). [41] Considering the initial G3.5-COONa solution has a pH value around 8.7, the exchange equilibrium between the anion (A) and the zwitterion (B) in Fig. S7 caused the peaks of H_a', b', c', d'_ broaden in ^1^H NMR spectrum. With the increase of TEMPO-COOH concentration, the pH value of the G3.5-COONa decreases to 6.0 and increased percent of the surface tertiary amines are protonated. The zwitterion (B) is the major form in the solution at high molar ratios of TEMPO-COOH and G3.5-COONa. Therefore, the peaks H_a', b', c', d'_ become narrow with the addition of TEMPO-COOH. This phenomenon is also observed when amino acids were titrated with acid. [42] In the case of interior methylene protons such as H_a–d_, the interior tertiary amine (pKa∼6.5) is not protonated at the beginning, and start to protonate after the addition of TEMPO, the exchange between protonated and non-protonated state caused the peaks H_a–d_ adjacent to these tertiary amine groups becoming broaden. Therefore, the linewidth of the peaks is controlled by exchange modulation of the tertiary amine groups rather than the PRE effect. To prove this speculation, we titrated G3.5-COONa dendrimer with acetic acid. As shown in Fig. 8, the same trend for linewidth of G3.5-COONa peaks was observed when this dendrimer was titrated with TEMPO-COOH and acetic acid. During this period, no PRE effect was observed between the protons (H_a'_, H_d'_) and the unpaired electron in the nitroxide radical (Fig. 8b). The failure of TEMPO-COO^−^ within G3.5-COONa cavities is probably due to the repulsion of TEMPO-COO^−^ ions and the anionic G3.5 surface.

## Conclusions

In this study, we investigated the host-guest chemistry of PAMAM dendrimers with amine, hydroxyl, and carboxylate surface functionalities and TEMPO radicals with amine, hydroxyl, and carboxyl groups using ^1^H NMR titration experiments. PRE effects were observed in TEMPO-COOH/G4-NH_2_ and TEMPO-COOH/G4-OH complexes. The inclusion complexes of TEMPO-COOH and G4-NH_2_ (G4-OH) are driven by ionic interactions rather than hydrophobic interactions. In other dendrimer/TEMPO systems, only protonation or deprotonation of the surface functionalities and interior tertiary amine groups occur. The absence of TEMPO-COOH encapsulation within G3.5-COONa is due to the repulsion of TEMPO-COO- anion and the negatively charged G3.5-COONa surface. This study proved that PRE NMR is a powerful method in the investigation of dendrimer-based host-guest interactions.

## Supporting Information

Figure S1 ^1^H NMR spectra of the G4-NH_2_/TEMPO-NH_2_ complexes in D_2_O/d6-DMSO solutions (80/20, V/V), the molar ratio of TEMPO-NH_2_ and G4-NH_2_ ranges from 0 to 64 (a). The linewidth variations of G4-NH_2_ peaks in ^1^H NMR spectra during the titration experiment (b).
(TIF)

Figure S2 ^1^H NMR spectra of the G4-OH/TEMPO-OH (a) and the G3.5-COONa/TEMPO-OH (c) complexes in D_2_O/d6-DMSO solutions (80/20, V/V), the molar ratio of TEMPO-OH and dendrimer ranges from 0 to 64. The linewidth variations of G4-OH and G3.5-COONa peaks in the ^1^H NMR spectra during the titration experiment are shown in (b) and (d), respectively.
(TIF)

Figure S3 Diffusion coefficients of G4-NH_2_ in the absence and presence of TEMPO-COOH at a molar ratio of 8 and 16 (a). Diffusion coefficients of G4-NH_2_ in the absence and presence of deoxycholate at a molar ratio of 32 (b). ^1^H NMR spectra of G4-NH2 before and after the addition of deoxycholate (c).
(TIF)

Figure S4 ^1^H NMR spectra of G4-NH_2_ titrated by acetic acid, the molar ratio of acetic acid and G4-NH_2_ ranges from 0 to 64.
(TIF)

Figure S5 ^1^H-^1^H NOESY of TEMPO-COOH derivative and G4-NH_2_ at a molar ratio of 32∶1. The mixing time is 300 ms.
(TIF)

Figure S6 (a) ^1^H NMR spectra of G4-OH titrated by acetic acid, the molar ratio of acetic acid and G4-NH_2_ ranges from 0 to 64. (b) The linewidth variations of G4-OH peaks (Hb') in ^1^H NMR spectra during the addition of TEMPO-COOH or CH_3_COOH.
(TIF)

Figure S7 The protonation and deprotonation equilibrium of tertiary amine groups and surface carboxylate groups of G3.5-COONa.
(TIF)

Abstract S1 **Graphical abstract.**
(DOC)

Text S1 The method of^ 1^H-^1^H NOESY spectrum of the TEMPO-COOH derivative and G4-NH_2_ complex.
(DOC)
