# Supplementary figures and images for: Paramagnetic NMR Investigation of Dendrimer-Based Host-Guest Interactions
Source: PLoS One. 2013 Jun 10;8(6):e64722. doi: 10.1371/journal.pone.0064722 (PMC3677888; doi:10.1371/journal.pone.0064722)

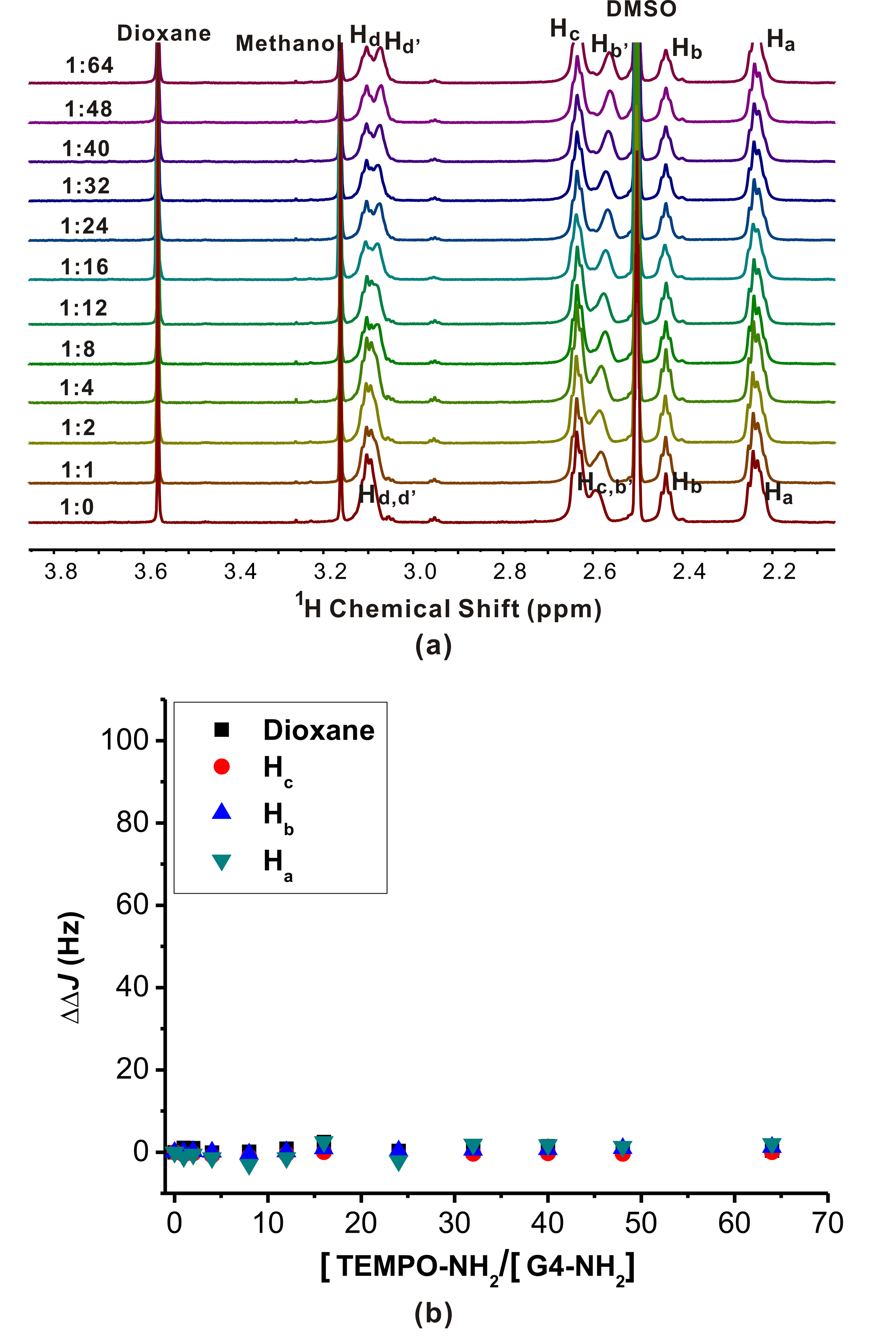

Supplement: Figure S1 — 1H NMR spectra of the G4-NH2/TEMPO-NH2 complexes in D2O/d6-DMSO solutions (80/20, V/V), the molar ratio of TEMPO-NH2 and G4-NH2 ranges from 0 to 64 (a). The linewidth variations of G4-NH2 peaks in 1H NMR spectra during the titration experiment (b). (TIF) [file pone.0064722.s001.tif]

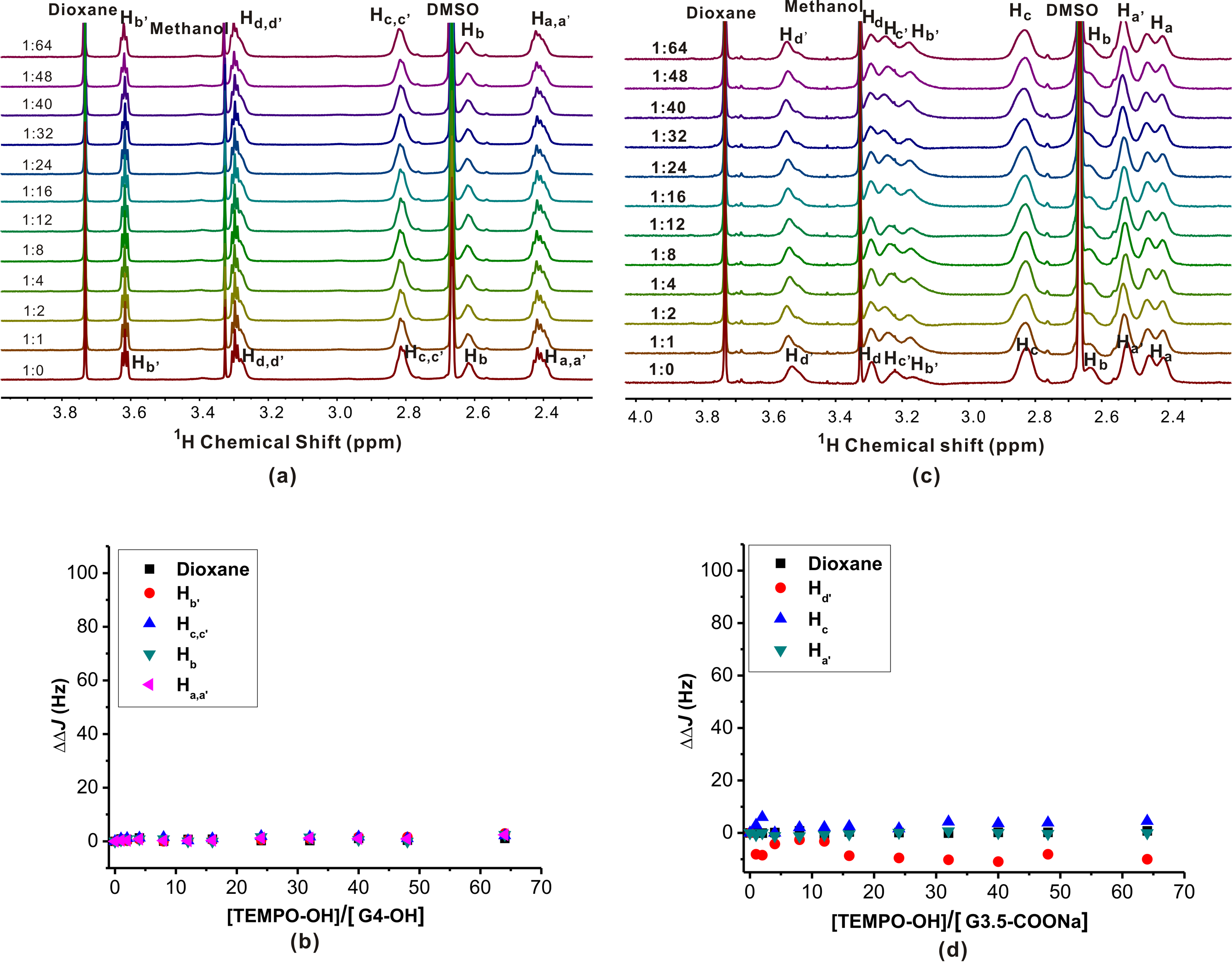

Supplement: Figure S2 — 1H NMR spectra of the G4-OH/TEMPO-OH (a) and the G3.5-COONa/TEMPO-OH (c) complexes in D2O/d6-DMSO solutions (80/20, V/V), the molar ratio of TEMPO-OH and dendrimer ranges from 0 to 64. The linewidth variations of G4-OH and G3.5-COONa peaks in the 1H NMR spectra during the titration experiment are shown in (b) and (d), respectively. (TIF) [file pone.0064722.s002.tif]

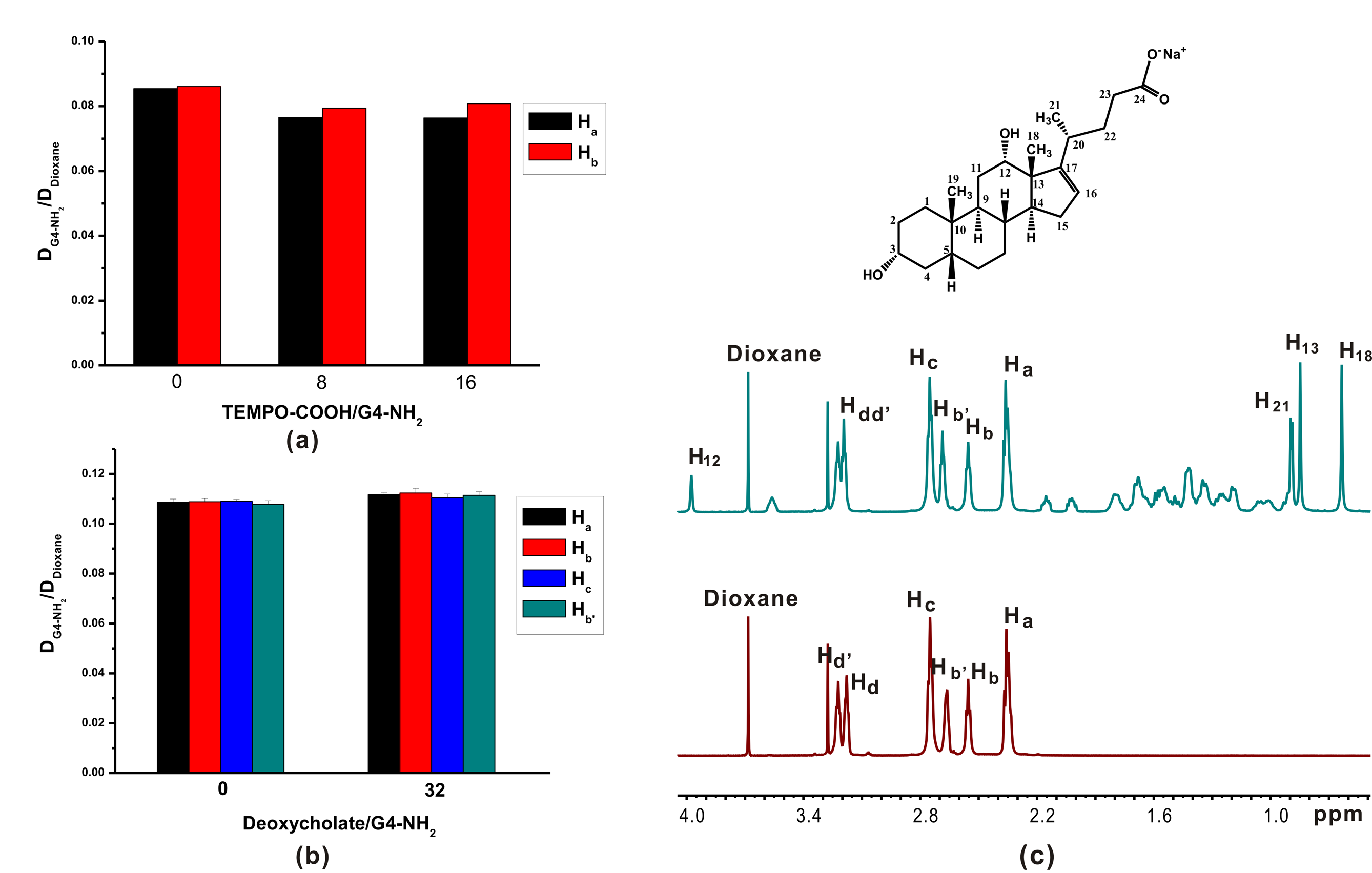

Supplement: Figure S3 — Diffusion coefficients of G4-NH2 in the absence and presence of TEMPO-COOH at a molar ratio of 8 and 16 (a). Diffusion coefficients of G4-NH2 in the absence and presence of deoxycholate at a molar ratio of 32 (b). 1H NMR spectra of G4-NH2 before and after the addition of deoxycholate (c). (TIF) [file pone.0064722.s003.tif]

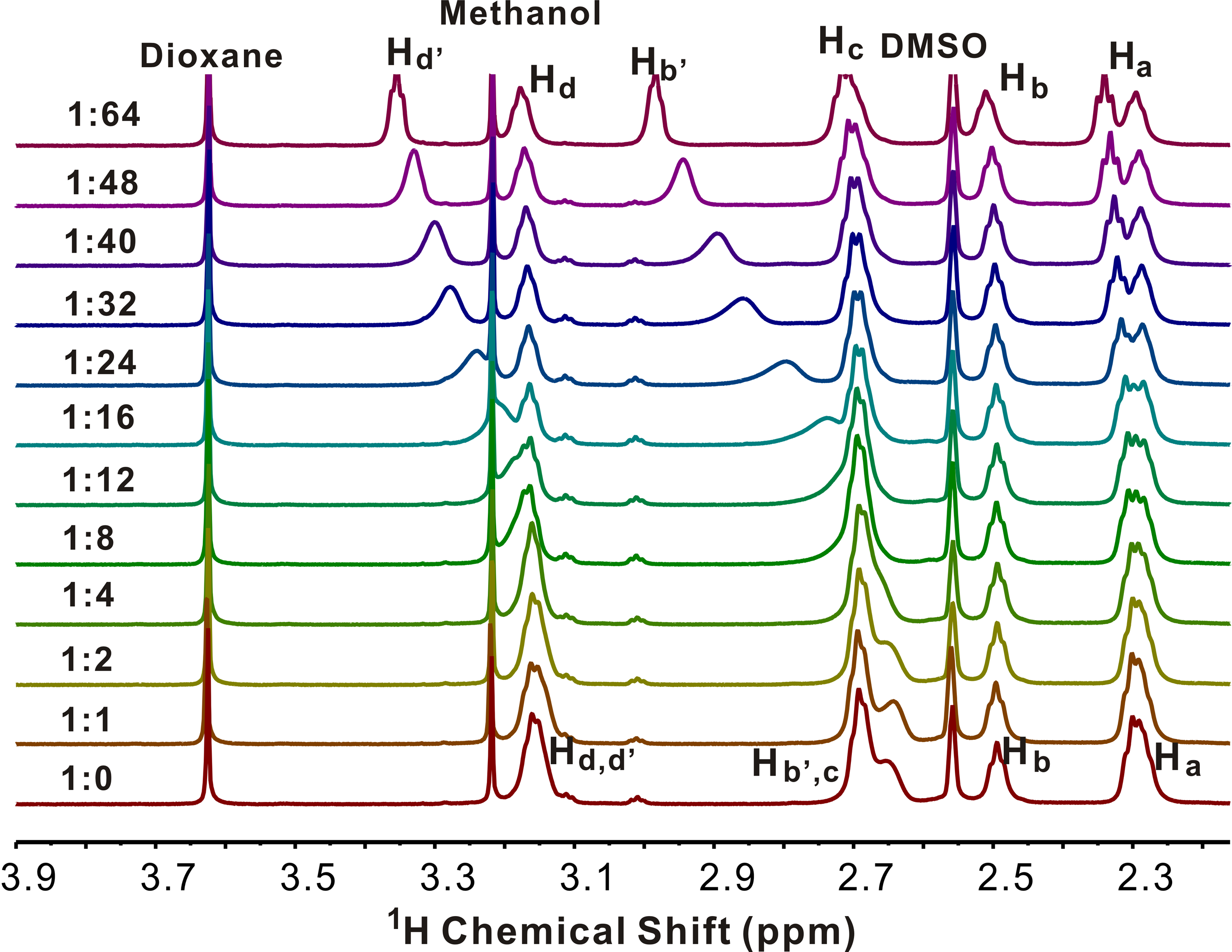

Supplement: Figure S4 — 1H NMR spectra of G4-NH2 titrated by acetic acid, the molar ratio of acetic acid and G4-NH2 ranges from 0 to 64. (TIF) [file pone.0064722.s004.tif]

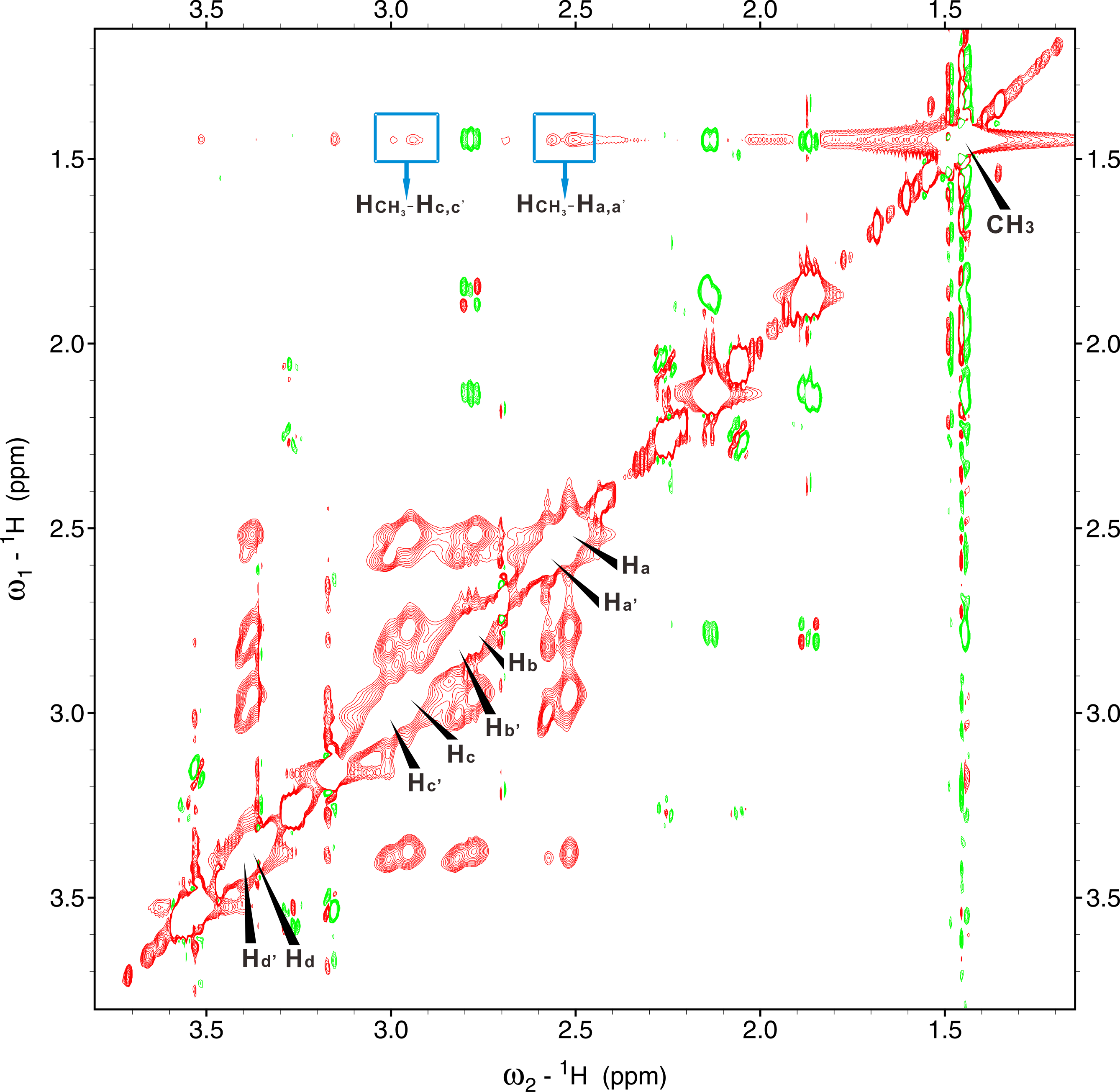

Supplement: Figure S5 — 1H-1H NOESY of TEMPO-COOH derivative and G4-NH2 at a molar ratio of 32∶1. The mixing time is 300 ms. (TIF) [file pone.0064722.s005.tif]

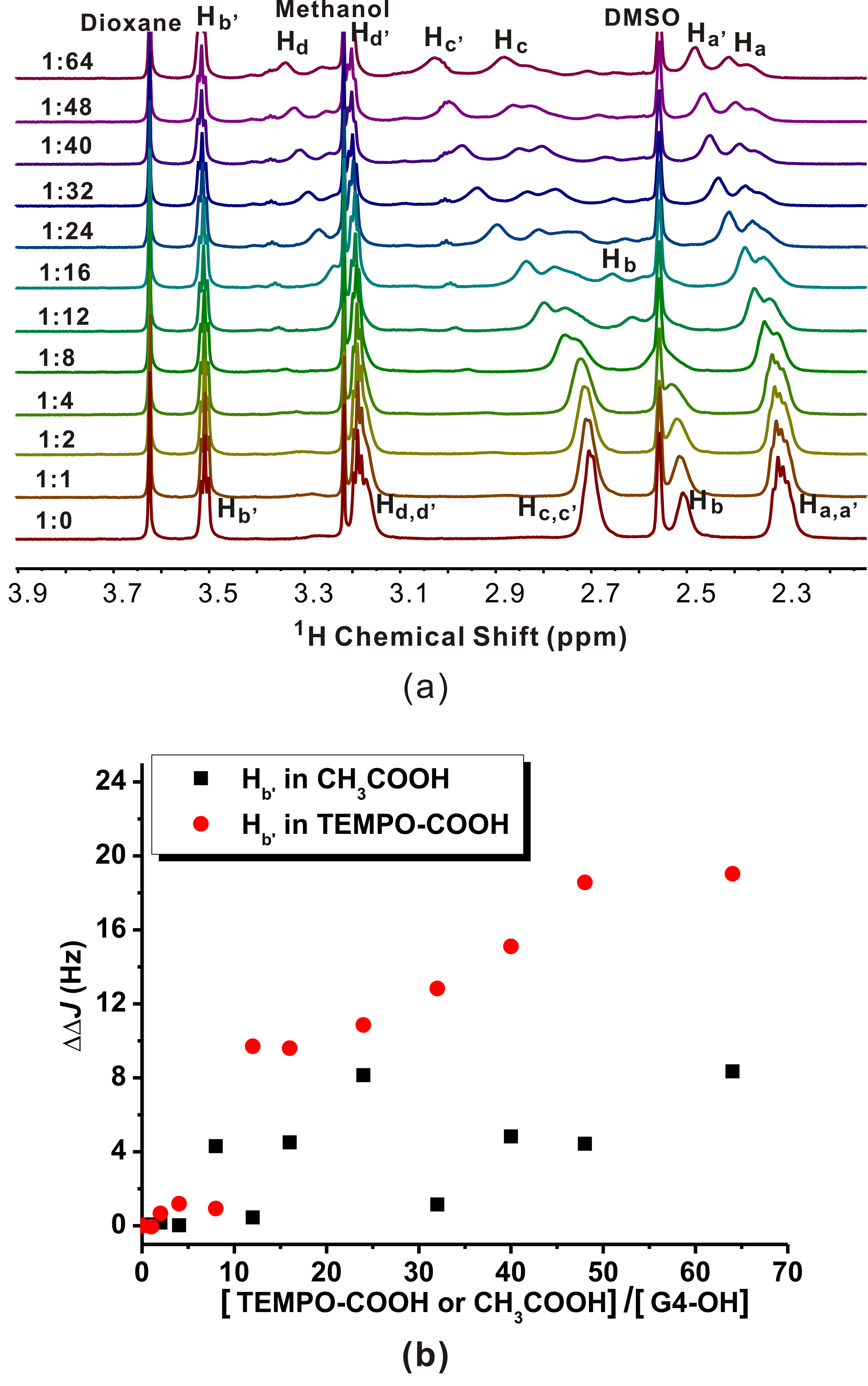

Supplement: Figure S6 — (a) 1H NMR spectra of G4-OH titrated by acetic acid, the molar ratio of acetic acid and G4-NH2 ranges from 0 to 64. (b) The linewidth variations of G4-OH peaks (Hb') in 1H NMR spectra during the addition of TEMPO-COOH or CH3COOH. (TIF) [file pone.0064722.s006.tif]

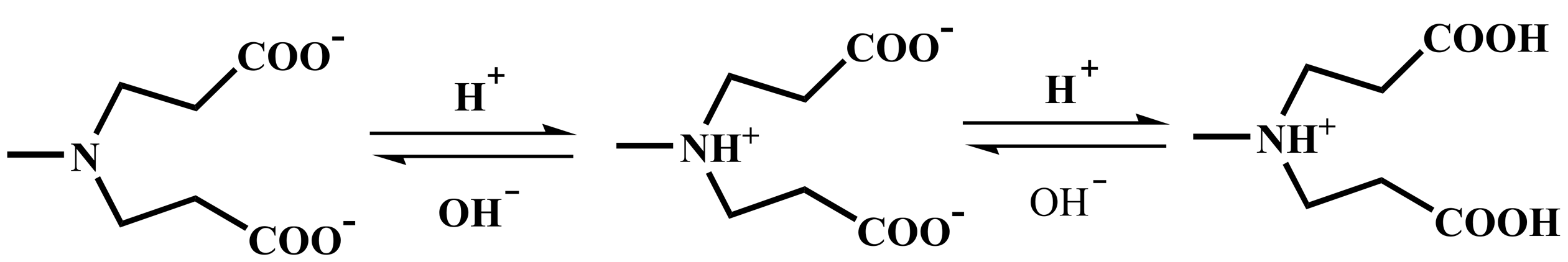

Supplement: Figure S7 — The protonation and deprotonation equilibrium of tertiary amine groups and surface carboxylate groups of G3.5-COONa. (TIF) [file pone.0064722.s007.tif]
