# Supplementary material for: Paramagnetic NMR Investigation of Dendrimer-Based Host-Guest Interactions
Source: PLoS One. 2013 Jun 10;8(6):e64722. doi: 10.1371/journal.pone.0064722 (PMC3677888; doi:10.1371/journal.pone.0064722)
Supplement: Abstract S1 — Graphical abstract. (DOC) [file pone.0064722.s008.doc]

**Graphical Abstract**

We reported the use of paramagnetic relaxation enhancement (PRE) NMR in revealing the host behaviors of PAMAM dendrimers with different surface functionalities. The results obtained in the present study provide new insights into dendrimer-based host-guest systems.
