# Supplementary material for: Paramagnetic NMR Investigation of Dendrimer-Based Host-Guest Interactions
Source: PLoS One. 2013 Jun 10;8(6):e64722. doi: 10.1371/journal.pone.0064722 (PMC3677888; doi:10.1371/journal.pone.0064722)
Supplement: Text S1 — The method of 1H-1H NOESY spectrum of the TEMPO-COOH derivative and G4-NH2 complex. (DOC) [file pone.0064722.s009.doc]

The ^1^H-^1^H NOESY spectrum of the TEMPO-COOH derivative and G4-NH_2_ complex at a molar ratio of 32:1 was obtained on a Varian 699.804 MHz instrument using standard pulse sequences at 298.2 ± 0.1 K in D_2_O. Generally, the ^1^H-^1^H NOESY experiments were performed with a 1 s relaxation delay, 146.63 ms acquisition time, and a 5.5 µs 90°pulse width. 300 ms was chosen as the mixing time for the optimization of cross-peak intensities with minimum distortions during the period for NOE establishment. 32 transients were averaged (256×1024). All the data were processed with NMRpipe software on a Linux workstation with standard Lorents-Gauss window function and zero-filling in both dimensions.
